# High-level production of L-threonine by recombinant *Escherichia coli* with combined feeding strategies

**DOI:** 10.1080/13102818.2014.927682

**Published:** 2014-07-10

**Authors:** Jian Wang, Li-Kun Cheng, Ning Chen

**Affiliations:** ^a^ Department of Bioengineering, Jilin University, Changchun, P.R. China; ^b^ Department of Bioengineering, Tianjin University of Science and Technology, Tianjin, P.R. China

**Keywords:** *Escherichia coli*, L-threonine, acetic acid, combined feeding strategy

## Abstract

The process of L-threonine production using *Escherichia coli* TRFC was investigated, and the result showed that there was a large amount of acetic acid in the broth. The effects of acetic acid, which is a known inhibitory metabolite in *E. coli* cultivation, on L-threonine production by recombinant *E*. *coli* TRFC were evaluated, and the result indicated that the growth of *E*. *coli* TRFC and L-threonine formation were significantly inhibited in the presence of acetic acid. Two combined feeding strategies were applied to L-threonine fed-batch fermentation in order to investigate the effects of the feeding strategy on L-threonine fermentation. The results showed that using the combined feeding strategy of pseudo-exponential feeding and glucose-stat feeding resulted in high cell density (36.67 g L^−1^) and L-threonine production (124.57 g L^−1^) as well as low accumulation of by-products. This work provides a useful approach for large-scale production of L-threonine.

## Introduction

L-threonine is an essential amino acid and is used as a supplement in animal feeds and human foods to improve their nutritional value, and also as a precursor of a number of commonly used flavouring ingredients.[1] The production of L-threonine depends mainly on direct fermentation by auxotrophic and regulatory mutants of *Escherichia coli*, *Serratia marcescens*, *Brevibacterium flavum* and *Corynebacterium glutamicum* using carbohydrates as substrates.[2] The *E*. *coli* mutants generated by mutagenesis or genetic manipulation are commonly chosen as producers due to their fast growth rates and well-known physiological characteristics.[3]

The mass production of L-threonine, due to its huge commercial demand, has been the focus of many research and development projects, e.g. microbial process development through strain improvement and fermentation process optimization.[4] A mutant *E. coli* HS3 strain in which aspartokinase was released from end-product inhibition was isolated and the growth medium components (glucose as a carbon source and corn steep liquor as the nitrogen source) were optimized to improve the mass production of L-threonine and reduce its cost.[5] A high production of threonine (80.2 g L^−1^) was achieved in an *E. coli* MT201 fed-batch culture system by adding biotin and oxygen-enriched air to basal minimal salt medium.[3] Okamoto achieved a final yield of 101 g L^−1^ of threonine in an industrially stable process for L-threonine fermentation with addition of D-, L-methionine and iron by an L-methionine autotrophic *E*. *coli* mutant.[5] In L-threonine production by *E. coli* TRFC strain, of various fermentation substrates and conditions investigated, sucrose was found to be the optimal initial carbon source, and glucose was selected as the optimal feeding medium.[1] Growth of *E*. *coli* on excess glucose under aerobic conditions causes the formation of acidic by-products, the most common of which is acetic acid.[6]

Acetic acid is undesirable because it retards growth and inhibits protein formation.[7] Moreover, acetic acid synthesis represents a diversion of carbon that might have otherwise been utilized for generation of biomass or the protein product.[8] High cell density cultivation (HCDC) techniques have been developed to enhance production of recombinant products. Decreasing acetic acid excretion is important for achieving HCDC of *E. coli*.[9] High cell density of *E. coli* can be achieved in fed-batch processes. Accumulation of acetic acid, which usually occurs during HCDC of *E*. *coli* and especially in the induction stage of protein synthesis during the production of the recombinant protein tumor necrosis factor-related apoptosis-inducing ligand (TRAIL), could be avoided with a combined strategy of exponential feeding and pH-stat feeding. High concentrations of biomass and active soluble TRAIL were obtained.[10] Another combined feeding strategy of pseudo-exponential feeding and glucose-stat feeding was used in L-tryptophan production, leading to high biomass and production of L-tryptophan due to high plasmid stability and low concentrations of acetic acid and glucose.[11] The effects of acetic acid on L-threonine production by *E. coli* and overcoming excretion of acetic acid in L-threonine production have not yet been systematically investigated so far. In this study, the effects of acetic acid on L-threonine production were investigated. To decrease the accumulation of acetic acid and increase the volumetric productivity of L-threonine, two combined feeding strategies were tested.

## Materials and methods

### Micro-organism and media

The L-threonine-producing *E*. *coli* strain TRFC (ILE^L^, AHV^r^) used in this study was obtained from the culture collection of Tianjin University of Science and Technology.[1] The culture was maintained on Luria–Bertani (LB) slant agar (5 g L^−1^ yeast extract, 10 g L^−1^ tryptone, 10 g L^−1^ NaCl and 2% agar).

The seed medium contained the following: 40 g L^−1^ of sucrose, 2 g L^−1^ of yeast extract, 1.5 g L^−1^ of KH_2_PO_4_, 10 g L^−1^ of (NH_4_)_2_SO_4_ and 0.5 g L^−1^ of MgSO_4_·7H_2_O. The medium for L-threonine fermentation contained the following: 70 g L^−1^ of sucrose, 2 g L^−1^ of yeast extract, 2 g L^−1^ of KH_2_PO_4_, 2 g L^−1^ of (NH_4_)_2_SO_4_, 0.5 g L^−1^ of MgSO_4_·7H_2_O, 0.1 g L^−1^ of FeSO_4_·7H_2_O and 0.1 g L^−1^ of MnSO_4_·7H_2_O. The pH of both seed and fermentation medium was adjusted to 7.0 with 4 mol L^−1^ NaOH.

### Culture methods

A 500 mL baffled flask containing 30 mL of seed medium was inoculated with a single colony of *E*. *coli* TRFC and cultivated at 36 °C for 12 h (200 r min^−1^). A 30 mL inoculum of this culture was added aseptically to a 5 L seed fermenter (Biotech-2002 Bioprecess Controller, Baoxing, Shanghai, China) containing 3 L of seed medium and cultivated at 36 °C for 12 h. The pH was adjusted to 7.0 with 25% (v/v) ammonia. The dissolved oxygen (DO) level was maintained at approximately 20% saturation by adjusting the agitation and aeration rates.

Fed-batch fermentation was performed in 30 L jar fermenters (Biotech-2002 Bioprecess controller). Thirty-litre jar fermenters containing 18 L of production medium were aseptically inoculated with culture developed in the seed fermenter (10% v/v). The temperature was maintained at 36 °C and the pH was adjusted to 7.0 with 25% (v/v) ammonia. The DO level was maintained at approximately 20% saturation by adjusting the agitation and aeration rates. When the initial carbon source was depleted, 80% (w/v) glucose solution was fed into the fermenter to meet specific experimental requirements. Different concentrations of acetic acid (0 g L^−1^, 1 g L^−1^, 2 g L^−1^, 3 g L^−1^, 5 g L^−1^) were added to the fermentation broth at the initiation of the fed-batch phase to determine the effects of acetic acid on L-threonine production. The pH of the acetic acid was adjusted to 7.0 with 4 mol L^−1^ NaOH.

### Analysis of fermentation data

DO, pH and temperature were measured automatically with electrodes attached to the fermenters. Dry cell weight was determined according to the method of Chen et al. [1] The concentration of glucose was determined using an SBA-40E biosensor instrument (Biology Institute of Shandong Academy of Sciences, China). The concentrations of L-threonine, alanine, valine and leucine were determined by high-pressure liquid chromatography (Agilent Technologies, Santa Clara, CA, USA). Acetic acid concentration was measured with a Bioprofile 300A biochemical analyser (Nova Biomedical, Waltham, MA, USA). The specific growth rate (μ) and specific production rate (π) were calculated as described previously.[11]

Data shown are averages of three independent experiments, with standard deviations.

## Results and discussion

### Process analysis of L-threonine production

Fed-batch fermentation of L-threonine was performed in a 30 L jar fermentor, and dry cell weight, concentrations of L-threonine and acetic acid were determined every two hours. The specific growth rate and specific production rate were also analysed.

**Figure 1.**
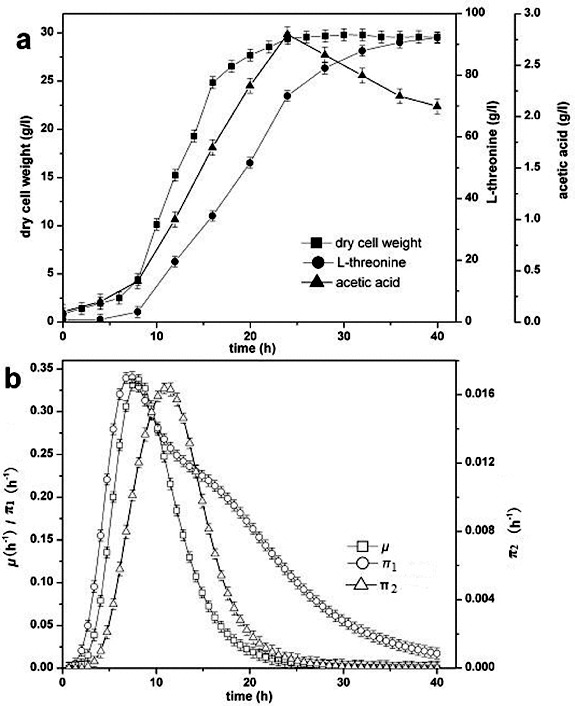
L-threonine production process under initial conditions. Specific production rate of L-threonine (π_1_); specific production rate of acetic acid (π_2_).

The process parameters of L-threonine production are presented in Figure 1. The kinetic analysis of strain growth showed that the exponential growth phase began 4 h after inoculation and the maximum specific growth rate was 0.3362 h^−1^. Cell growth increased until 24 h and reached a dry cell weight of 29.42 g L^−1^. L-threonine production increased with cell growth and reached 92.18 g L^−1^ at the end of the cultivation period. The accumulation of acetic acid began 2 h after inoculation, and the maximum concentration of acetic acid was 2.81 g L^−1^ at 24 h. Both the specific L-threonine production rate and specific acetic acid production rate were high during the early fermentation phase because the biomass was relatively low. The maximum specific production rate of L-threonine and acetic acid were 0.3401 h^−1^ and 0.01637 h^−1^, respectively.

Two primary causes for excretion of acetic acid are that strain respiration is restricted because of inadequate oxygen supply and glucose uptake rate surpasses the turnover capacity of the tricarboxylic acid cycle (TCA cycle).[12] DO level is an important parameter for formation of product. The Crabtree effect occurs with a high DO level and acetic acid is excreted at a low DO level. The DO level also affects the metabolic flux distribution. Not only does the oxygen supply satisfy the strain growth to avoid the excretion of acetic acid, but also more L-threonine is synthesized with maintaining the DO level at 20%.[13] Aerobic acetogenesis in *E. coli* has been described as an ‘overflow’ metabolism under adequate oxygen conditions. In this explanation, acetic acid excretion is the result of imbalance between carbon influx into the central metabolism and the limited capacity of the TCA cycle or respiration.[14] Two acetic acid synthesis pathways, phosphotransacetylase-acetate kinase (Pta-AckA) and pyruvate oxidase (PoxB), contribute to the synthesis at the beginning of the overflow metabolism, i.e. the onset of acetate excretion.[15]

### Effect of acetic acid on L-threonine fermentation

In order to determine the effects of acetic acid on L-threonine production, different concentrations of acetic acid were added to the fermentation broth at the initiation of the fed-batch phase. The specific growth rate was controlled at 0.1 h^−1^ with an exponential feeding strategy to avoid the accumulation of acetic acid in the fermentation process.

The effects of acetic acid on L-threonine production are shown in Table 1. The maximum accumulated concentration of acetic acid was only 0.014 g L^−1^ when the specific growth rate was controlled at 0.1 h^−1^. The dry cell weight, production of L-threonine and specific growth rate decreased with increasing the concentrations of acetic acid supplemented to the medium. This indicated that acetic acid inhibited the growth of strain *E*. *coli* TRFC as well as the formation of L-threonine. The fermentation period was prolonged because of the addition of acetic acid. Thus, it was crucial to reduce the excretion of acetic acid to increase the dry cell weight and production of L-threonine.

**Table 1.** Comparison of parameters in L-threonine production at different concentrations of supplemental acetic acid.

	Concentrations of supplemental acetic acid (g L^−1^)				
Kinetic parameters	0	1	2	3	5
Dry cell weight (g L^−1^)	25.67	25.07	23.35	22.72	21.46
L-threonine (g L^−1^)	82.35	80.25	76.56	71.47	62.42
Specific growth rate (h^−1^)	0.100	0.097	0.092	0.089	0.085
Incremental acetic acid (g L^−1^)	0.014	0.013	0.014	0.014	0.013
Fermentation period (h)	40	42	45	52	55

### Application of feeding strategies in L-threonine production

#### Dry cell weight, production of L-threonine and carbon source concentration

*E*. *coli* produces acetic acid as an extracellular by-product of aerobic fermentation when the specific growth rate exceeds the value of the threshold growth rate. According to the demand of specific growth rate and glucose concentration, a combined feeding strategy of exponential feeding and pH-stat feeding, and another strategy of combined pseudo-exponential feeding and glucose-stat feeding, were used in L-threonine production. In L-tryptophan production by *E. coli*, high dry cell weight and production of desired product were obtained with the maximum specific growth rate below 0.25 h^−1^ due to low excretion of acetic acid and high plasmid stability.[11] Plasmid instability is one of the reasons for the decreased specific yield.[16] In our experiments, the specific growth rate of the L-threonine-producing *E*. *coli* strain was controlled below 0.25 h^−1^ and the concentration of residual glucose was maintained at a low level with the two combined feeding strategies.

**Figure 2.**
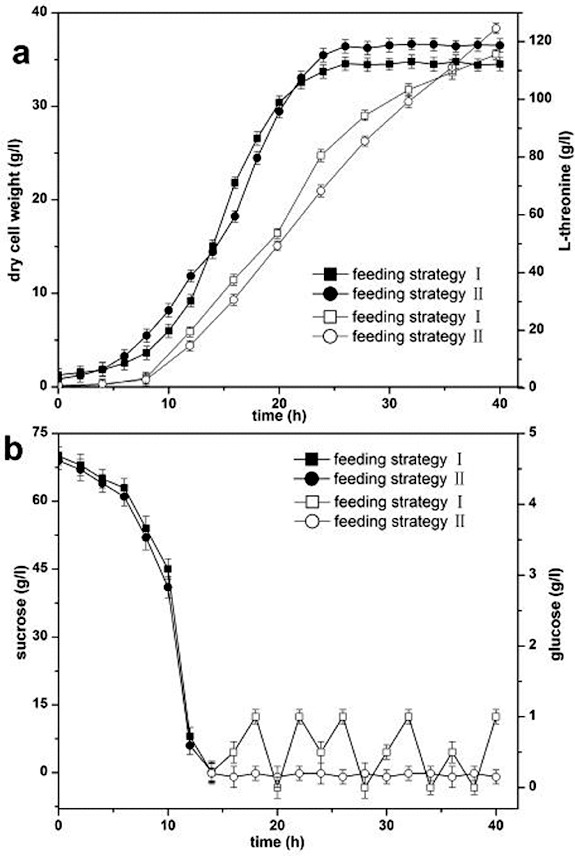
Dry cell weight, production of L-threonine and carbon source concentration in L-threonine fermentation. Feeding strategy I: combined feeding strategy of exponential feeding and pH-stat feeding; feeding strategy II: combined feeding strategy of pseudo-exponential feeding and glucose-stat feeding. (a) Dry cell weight (filled symbols) and production of L-threonine (open symbols). (b) Concentration of sucrose (filled symbols) and concentration of glucose (open symbols).

The results from the fermentation with the two combined feeding strategies are presented in Figure 2. The concentration of residual glucose was maintained at 0–1 g L^−1^ with the combined feeding strategy of exponential feeding and pH-stat feeding, and was maintained at approximately 0.15 g L^−1^ by using the combined feeding strategy of pseudo-exponential feeding and glucose-stat feeding after the initial carbon source was depleted at 14 h. The dry cell weight (36.58 g L^−1^) and production of L-threonine (124.57 g L^−1^) were improved by using the combined feeding strategy of pseudo-exponential feeding and glucose-stat feeding. Low substrate concentrations were beneficial for achieving optimal growth conditions.[17]

#### Accumulation of by-products

**Figure 3.**
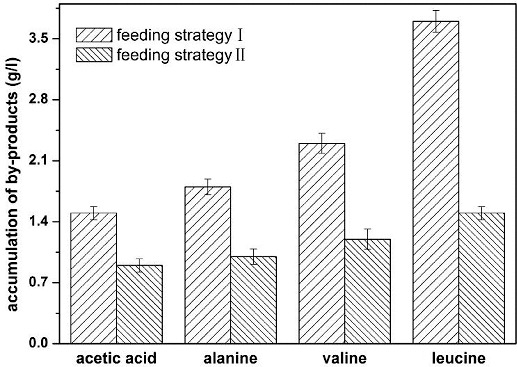
Accumulation of by-products with different feeding strategies. Feeding strategy I: combined feeding strategy of exponential feeding and pH-stat feeding; feeding strategy II: combined feeding strategy of pseudo-exponential feeding and glucose-stat feeding.

The accumulation of by-products with the two combined feeding strategies is shown in Figure 3. The concentration of by-products with the combined feeding strategy of pseudo-exponential feeding and glucose-stat feeding were lower than that with the combined feeding strategy of exponential feeding and pH-stat feeding. The accumulation of acetic acid was 0.9 g L^−1^ using the combined feeding strategy of pseudo-exponential feeding and glucose-stat feeding. Acetic acid excretion is not observed when the specific growth rate of *E*. *coli* is lower than a certain threshold, due to carbon limitation in continuous cultures or the quality of carbon source in batch cultures.[14] The energy produced by oxidative metabolism can satisfy the demand of biosynthesis and catabolism with a low specific growth rate; thus, no acetic acid was accumulated. However, the energy produced only by oxidative metabolism is insufficient with a high specific growth rate, so adenosine triphosphate (ATP) and nicotinamide adenine dinucleotide (NADH) should be stored by the pathway of acetic acid production.[18] In this context, aerobic acetogensis of *E*. *coli* has been proposed as a means of generating extra ATP to support faster growth.[19] Acetic acid concentration was kept at zero during the entire cultivation period due to the low glucose concentration, and both the cell density and phenylalanine concentration were increased markedly.[20] When the specific growth rate of *E*. *coli* TRFC was controlled at 0.10 h^−1^ in L-threonine production, a very small amount of acetic acid was excreted.

Apart from acetic acid, some other by-products were also accumulated in L-threonine production, such as alanine, valine and leucine. Using the combined feeding strategy of pseudo-exponential feeding and glucose-stat feeding, the accumulation of L-alanine, L-valine and L-leucine were up to 1.0 g L^−1^, 1.2 g L^−1^ and 1.5 g L^−1^, respectively, which were lower than those with the combined feeding strategy of exponential feeding and pH-stat feeding. It was reported that less by-products were accumulated due to a lower metabolic flux of the TCA cycle and low glucose concentration, and high glucose conversion rate was obtained with a higher metabolic flux of the hexose monophosphate pathway (HMP) and less accumulation of by-products.[8, 13] The results showed that more carbon source entered the HMP pathway and less carbon source entered the Embden–Meyerhof–Parnas pathway (EMP) and the TCA cycle with the combined feeding strategy of pseudo-exponential feeding and glucose-stat feeding.

## Conclusions

In conclusion, the combined feeding strategy of pseudo-exponential feeding and glucose-stat feeding of L-threonine production with *E*. *coli* strain TRFC resulted in very efficient L-threonine production with low by-product formation. Final acetic acid, L-alanine, L-valine and L-leucine concentrations were limited to 0.9 g L^−1^, 1.0 g L^−1^, 1.2 g L^−1^ and 1.5 g L^−1^, respectively, thus reducing the efforts in downstream processing. The presented process for L-threonine production will be a promising alternative to present industrial production and should reduce production costs significantly.
